# Blood and hair mercury concentrations among Cree First Nations of *Eeyou Istchee* (Quebec, Canada): time trends, prenatal exposure and links to local fish consumption

**DOI:** 10.1080/22423982.2018.1474706

**Published:** 2018-05-22

**Authors:** Susannah Ripley, Elizabeth Robinson, Louise Johnson-Down, Anne Andermann, Pierre Ayotte, Michel Lucas, Evert Nieboer

**Affiliations:** a Department of Epidemiology, Biostatistics and Occupational Health, McGill University, Montréal, Québec, Canada; b Public Health Department, Cree Board of Health and Social Services of James Bay, Mistissini, Québec, Canada; c School of Dietetics and Human Nutrition, McGill University, Sainte Anne de Bellevue, Québec, Canada; d Department of Family Medicine, McGill University, Montréal, Québec, Canada; e Department of Social & Preventive Medicine, Laval University, Québec, QC, Canada; f Axe Santé des populations et pratiques optimales en santé, Centre de recherche du CHU de Québec - Université Laval, Québec, QC, Canada; g Quebec Toxicology Centre, Institut National de Santé Publique du Québec, Québec, QC, Canada; h Department of Biochemistry and Biomedical Sciences, McMaster University, Hamilton, ON, Canada

**Keywords:** Methylmercury, pregnant women, women of childbearing age, fish consumption, exposure assessment

## Abstract

To describe exposure to methylmercury among Cree, focusing on women of childbearing age, we used data from 2 studies. Multiple regression was employed to examine associations between blood and hair mercury concentrations and consumption of locally harvested fish.

Approximately 9.9% of non-pregnant women aged 15–44 y and 3.9% of pregnant women required follow-up according to Health Canada’s blood mercury guidance value of 40 nmol/L. 8% of hair mercury observations in the non-pregnant women and 2.5% among pregnant women exceeded the equivalent threshold of 10 nmol/g. The geometric mean blood mercury concentration was 12.7 nmol/L in 1,429 persons aged 8 and over, and 17.7 nmol/L in adults aged 18 and older. The proportion of hair mercury concentrations greater than 12.5 nmol/g decreased in all age-sex groups when comparing the 2002–2009 data to published values for 1993–1994. Among women of childbearing age, local fish consumption was associated with increased blood and hair mercury concentrations.

While over 90% of women of childbearing age in this population have acceptable levels of mercury, ongoing intake of mercury suggests that their consumption of fish with known high mercury content be minimised. Reducing consumption of fish known to be high in mercury content needs to be balanced with promoting ongoing connection to Cree culture and land-based activities that are also important determinants of health.

## Introduction

Since the first studies in the 1970s, mercury exposure has been an ongoing concern among the Cree First Nations of *Eeyou Istchee* in northern Quebec [1]. An initial ban on local fish consumption was greeted with anger, sadness and disbelief [2]. Subsequent studies by the Grand Council of the Crees revealed that mercury levels in fish depended on fish species, size and geographic location, and advisories were modified accordingly.

With a total population of 17,800 in 2016 [3], the Cree live in 9 communities located between the 49th and 56th parallels east of James and Hudson Bays in northern Quebec, Canada. The physical environment of the James Bay Cree traditional territory includes the Hudson Plains, Boreal Shield and Taiga Shield ecozones [4].

Some early cases of exposure to high levels of mercury in Canada were associated with eating fish harvested downstream from chlor-alkali paper plants [5]. Even in isolated northern areas, fish and marine mammals were found to contain high levels of mercury due to atmospheric fallout of mercury from industrial sources outside the watershed [6]. Extensive hydroelectric development in the James Bay Region in the 1980s also contributed to the elevation of methylmercury concentrations in fish [5]. In this context, the reservoirs constructed enhanced microbial decomposition of organic matter, thereby increasing the rate of methylation of mercury already present in the environment [7]. This biologically available mercury then accumulates in fish, a staple food in the traditional diet of the Cree.

Exposure to mercury has known negative effects, especially when the exposure occurs prenatally. The Nunavik Child Development Study, a cohort study in the Inuit of northern Quebec, demonstrated that prenatal methylmercury exposure is associated with neurological problems in young children, including poorer immediate and long-term memory [8,9] as well as an increase in symptoms of attention deficit/hyperactivity disorder [10]. In the same cohort at a mean age of 11 years, prenatal methylmercury exposure was associated with lower estimated IQ [11]. A meta-analysis of 3 studies of fish-eating populations in the Seychelles, the Faroe Islands and New Zealand found a linear decrease of 0.18 IQ points associated with every part per million of mercury in maternal hair [12]. Recent publications have shown that blood/hair mercury levels in the Cree remain elevated in comparison with other Canadian populations [13,14]. Nonetheless, preliminary analyses suggest that median mercury levels in the James Bay Cree are still well below levels of concern [15], and that mercury levels have decreased greatly in comparison to the 1990s [15], but evidence for these changes has not been statistically demonstrated.

In order to minimise exposure to methylmercury, educational campaigns have advised the James Bay Cree to reduce consumption of fish species known to have higher mercury levels, such as pike, walleye, lake trout and burbot [5]. However, hunting and eating fish is important to the traditional Cree way of life and has many health benefits. A shift in the diet in past decades from traditional foods to market foods has been associated with lower food quality and nutrient intake [16], with a consequent rise in the prevalence of obesity and chronic diseases [13]. Recent recommendations [17] promote the consumption of fish and other traditional foods for their health benefits and importance to the Cree lifestyle and culture. Cree traditional foods have cultural significance because the activities of harvesting, preparing and eating traditional foods are social activities; they bring families and communities together and thereby strengthen indigenous identity [18].

Women who are pregnant or may become pregnant face a tradeoff between the benefits and risks of fish consumption [19]. In Nunavik (northern Quebec), high cord blood levels of the omega 3 fatty acid docosahexaenoic acid (DHA) found in fish and marine mammals, were associated with improved memory and visual processing as well as neurobehavioural outcomes of school-age children despite relatively high PCB-153 in cord plasma, and of lead and mercury in cord blood [20]. Confounding by prenatal DHA attenuated the observed relationship between prenatal mercury exposure and IQ [11]. Several investigations of populations with moderate fish consumption have suggested benefits from moderate fish intake [21]. However, a prospective cohort study of mother–child pairs in Massachusetts, USA found no evidence for a beneficial or harmful effect of prenatal fish consumption, mercury exposure or plasma n-3 fatty acids on children’s cognitive function [21].

In 2010, methylmercury blood guidance values for Canada were published [22]. For children under 18 y and women of childbearing age, no follow-up was required for blood levels under 40 nmol/L, which is equivalent to 10 nmol/g in hair [13]. Follow-up testing within 6 months and dietary counselling were recommended for women aged 18–49 y and children under 18 y who had measurements between 40 and 200 nmol/L in blood. Immediate re-testing and the scheduling of an appointment with a public health professional to review exposure sources were recommended for individuals with blood levels above 200 nmol/L [22]. These recommendations also applied to women with hair mercury measurements at 10–50 nmol/L and over 50 nmol/L, respectively. The thresholds below which no follow-up was required were arrived at by first deciding on a benchmark dose (a mercury level at which there is a 5% excess risk of negative child neurodevelopmental outcomes, according to epidemiological studies). The lower bound of the 95% confidence interval of the benchmark dose was then divided by a safety or uncertainty factor of 5 [22].

Given the health benefits of fish and the cultural importance of traditional Cree foods, there is a need for updated knowledge about both the state of mercury exposure and its relationship with locally caught fish consumption in this population. We report the proportion of pregnant and childbearing-aged Cree women in *Eeyou Istchee* that is at increased risk due to mercury exposure. A second objective is to verify that there has been a decrease in mercury exposure among the James Bay Cree from the mid-1990s to the present. Furthermore, we describe the relationship between consumption of fish caught locally and mercury exposure in order to evaluate the balance of risks and benefits of its consumption among this population. We also address how our results might inform public health actions, such as decisions about the continuation of screening, and the issuing of food consumption advisories aiming to reduce any potential impacts of mercury while encouraging traditional dietary practices.

## Methods

### Data sources

Our data have 2 main sources, namely a cross-sectional research study and an administrative data set collected as part of prenatal care provided by the Cree Board of Health and Social Services of James Bay (CBHSSJB). The cross-sectional *Nituuchischaayihtitaau Aschii* Multi-Community Environment-and-Health (E&H) Study included participants from the 9 Cree First Nation communities located in the *Eeyou Istchee* territory. A total of 2 communities were sampled in the summer of 2002 in an effort to assess potential health concerns about past mine operations located near one of the communities [23], and data from this survey were merged with those for the other 7 communities obtained during the summer months of 2005–2009 [24]. The second data source was a prenatal screening programme conducted by the CBHSSJB during the 2006–2011 period as part of its Maternal-Infant Health Program (MIHP). In addition, hair mercury concentration data were extracted from the 1998 study by Dumont et al. [1] to allow a comparison of exposure to mercury by the James Bay Cree in the 1990s and the E&H Study period.

### Study populations

Details on the recruitment and participation in the E&H Study are available elsewhere (for links see [24,25]). Briefly, eligibility was determined by registry in the *Eeyou* Beneficiaries List of the James Bay and Northern Quebec Agreement, a government register maintained by the *Ministère* de La *Santé et* Des *Services Sociaux* Du *Québec (MSSS)*. Pregnant women were excluded from this study. Potential participants in each community were selected by random sampling without replacement from the list of beneficiaries. Participants were stratified by age groups (0–8 y, 8–14 y, 15–39 y, and 40 y and over), by sex, and by community of residence in order to achieve adequate population representation. Ethics approval was granted by the research ethics committees of the Université Laval, McGill University, and the CBHSSJB, in partnership with McMaster University. Written informed consent was obtained from all participants or their guardians.

### Protocols

As described previously [26,27], hair and blood samples were collected and analysed for total mercury content. Hair strands from the occipital region of the head were cut close to the scalp and the 0–2 cm segment was analysed. The limit of detection (LD) for mercury in hair was 0.1 nmol/g [26]. Mercury concentrations below the LD were substituted with LD/2: specifically, 0.05 nmol/g in hair and 0.3 nmol/L in blood.

In the E&H Study, questionnaires were administered to gather anthropometric, clinical and lifestyle data, as well for assessment of consumption frequencies of market and traditional foods. The complete set of questionnaires are available online (see Appendix 1 of [24]), and included individual, clinical, 24 h dietary recall, and market and traditional food frequency questionnaires. The latter asked participants to report the frequency of having eaten various traditional foods (various species and body parts of fish, game and fowl, as well as berries) during each season for the past 12 months. In the present analysis, fish were divided into 2 categories: high mercury fish and lower mercury fish (for a full list of the food items in each category; see Supplementary Table S1). Game and fowl were not included in the analysis. The Cree do not eat marine mammals, and consumption of game and fowl known to have higher levels of mercury (such as loon and otter) is rare. The high mercury fish group was composed of 4 predatory fish species that are known to have high mercury concentrations, namely Walleye (*Sander vitreus*), Pike (*Esox lucius*), Burbot (*Lota lota)* and Lake Trout (*Salvelinus namaycush*) [17]. Our study did not address the consumption of market foods that might have constituted additional sources of methylmercury, such as canned fish.

The MIHP screening was integrated into the usual prenatal care protocols of the CBHSSJB. All pregnant women were asked to provide a blood and hair sample for mercury at the first prenatal visit (around 8–10 weeks of gestation). Verbal informed consent was obtained by the nurse who collected blood and hair samples (in some cases, the latter was taken by a Community Health Representative). The analytical protocols for mercury were as employed in the E&H Study.

### Exceedance of guidelines

Proportions and means constituted the mercury exposure measures. The Bonferroni’s procedure was applied to correct for multiple testing [28]. Mercury concentrations in hair and blood had a log-normal distribution and geometric mean values are reported. Significance tests were conducted at the α = 0.05 level and 95% confidence intervals.

At the time of the E&H Study, the concern ranges of 60–100 nmol/L (blood) and 20–30 nmol/g (hair) were adopted for women of childbearing age, pregnant women and children under 15 y (see Appendix 6 in [21]). Women with blood or hair mercury concentrations in the above ranges were offered dietary counselling to limit their exposure. Those having concentrations in the action range (>100 nmol/L in blood and >30 nmol/g in hair) were referred to a physician for follow-up. In 2010, new methylmercury blood guidance values were published [22]. For children under 18 y and women of childbearing age, these guidance values for the concern level were 40–200 nmol/L and an action level of >200 nmol/L in blood [22], respectively equivalent to 10–50 nmol/g and >50 nmol/g in hair [13]. Follow-up testing within 6 months and dietary counselling were recommended for women aged 18–49 y and children under 18 y who had measurements in the concern range, while immediate re-testing and the scheduling of an appointment with a public health professional to review exposure sources were recommended for individuals in the action range [22].

Indirect standardisation was performed to compare the prevalence rates of high mercury exposure between the E&H women and MIHP participants aged 15–44. The rate of high mercury exposure was taken as the proportion of women in each age group with blood or hair mercury concentrations in the Health Canada concern and action ranges.

### Comparisons with past levels

For comparisons with the past, data were drawn from Table 2 in Dumont et al. (1998) [1]. Mercury exposure was measured only in hair samples in the 1993–1994 survey, with 12.5 nmol/g the LD [1]. Comparison of means between these time periods is therefore difficult because the LD of total mercury in hair decreased substantially to 0.1 nmol/g in the current study [1,26]. Consequently, we compared the proportion of the population having hair mercury concentrations above 12.5 nmol/g in these 2 data sets. A conversion factor of 4.985 was used to convert mass units (µg/g) to SI units (nmol/g).

**Table 1.** Geometric mean and range of mercury concentrations in James Bay Cree women aged 15–44 and proportion of these women who exceeded Health Canada’s 2010 guidelines for blood and hair mercury concentrations.

	Blood sampling		Hair sampling	
	E&H Study non-pregnant women 2002–2009 (N = 516)	MIHP pregnant women 2006–2011 (N = 1374)	E&H Study non-pregnant women 2002–2009 (N = 510)	MIHP pregnant women 2006–2011 (N = 913)
***Mean age (y)^a^***	28.8	25.7	28.7	25.5
***Mercury concentration range (minimum, maximum)***	0.3, 170.0 nmol/L	0.2, 180.0 nmol/L	0.05, 57.7 nmol/g	0.05, 44.1 nmol/g
***Mercury concentration*** ***(geomean ± 95% CI)***	9.1 (8.2, 10.1) nmol/L	4.4 (4.1, 4.7) nmol/L	1.4 (1.3, 1.6) nmol/g	0.84 (0.77, 0.91) nmol/g
***Proportion (% ± 95% CI)***				
*No follow-up required^b^*	90.1 (87.1, 92.5)	96.1 (94.9, 97.0)	92.0 (89.2, 94.1)	97.5 (96.2, 98.4)
*Concern range^c^*	9.9 (7.5, 12.9)	3.9 (3.0, 5.1)	7.8 (5.7, 10.6)	2.5 (1.6, 3.8)
*Action range^d^*	0	0	0.2 (0.0, 1.2)	0

^a^Mean mercury concentrations and proportions presented in this table are not age-standardised. ^b^<40 nmol/L in blood, <10 nmol/g in hair; ^c^40–200 nmol/L in blood, 10–50 nmol/g in hair; and ^d^>200 nmol/L in blood, >50 nmol/g in hair.

**Table 2.** Proportion of hair mercury concentrations above 12.5 nmol/g (2.5 μg/g) in the James Bay Cree of northern Quebec, comparing results from the *Nituuchischaayihtitaau Aschii* Environment-and-Health Study (2002–2009) and earlier estimates by Dumont et al. [1].

	1993/94^a^		2002-2009^b^		
Sex-Age group (y)	N	Per cent above the LOD of 12.5 nmol/g	N	Per cent above 12.5 nmol/g	Difference in % above 12.5 nmol/g (Bonferroni-adjusted 95% CI) for 1993/94–2002/09
*Females*					
*4–14*	582	5.3	191	1.0	4.3 (0.8, 7.6)
*15–39*	850	13.3	455	4.2	9.1 (5.0, 13.2)
*≥40*	492	66.5	228	40.8	25.7 (15.0, 36.3)
*Males*					
*4–14*	503	7.6	209	1.0	6.6 (2.7, 10.5)
*15–39*	742	21.2	241	8.3	12.9 (6.5, 19.3)
*≥40*	430	71.4	176	43.2	28.2 (16.4, 40.0)

^a^Dumont et al. [1].

^b^*Nituuchischaayihtitaau Aschii* Environment-and-Health Study

### Relationship between locally caught fish consumption and mercury exposure

Fish were classified as high mercury fish or low mercury fish as presented in supplementary Table S1. Annual consumption frequencies were calculated by summing the mean daily consumption frequencies in each of the 4 seasons.

Multiple linear regression was employed to evaluate the relationship between blood and hair mercury concentrations and fish consumption. The dependent variable in each regression was the natural log-transformed mercury concentration (in blood or in hair, respectively) with the consumption of the 2 categories of fish as the predictor variables. Since the latter had high 0 consumption frequencies, frequency of consumption was classified into 3 levels for each category of fish.

All analyses were conducted using the statistical software R (R version 3.4.0, R Foundation for Statistical Computing, Vienna, Austria).

## Results

Of those invited (n = 3588), 48% participated in the E&H Study (n = 1730), with the invited/population ratio ranging 0.18–0.32 for the largest communities and 0.39–0.53 for the smallest. Blood and hair samples for mercury analysis were provided by 1430 and 1620 participants, respectively. Blood samples were provided by individuals aged 8 and over, while hair sampling involved all age groups. For women of childbearing age (15–44 y), the number of participants was 516 (blood) and 510 (hair). For men and women aged 8 y and over the geometric mean mercury concentrations were 12.7 nmol/L in blood and 2.2 nmol/g in hair, while for adults aged 18 y and over the geometric mean mercury concentrations were 17.7 nmol/L in blood and 3.1 nmol/g in hair.

In the MIHP screening component, 1,374 pregnant women gave blood samples and 913 provided hair samples. Participation rates were calculated as the number of women who consented to donate blood or hair samples divided by the number of mothers who gave birth during the 2006–2011 period according to the *MSSS* birth registry. The participation rates were 58.1% (blood) and 38.5% (hair). When the study sample was limited to women aged 15–44 y, 11 blood samples and 4 hair samples were excluded because the participants were younger than 15 y.

### Exceedance of guidelines

Exceedances of mercury concentration guidelines in blood and hair are summarised in Table 1. Using the 2010 guidance values, 90.1% of non-pregnant women and 96.1% of pregnant women had blood mercury concentrations below the lower cutoff, while for hair mercury concentrations these proportions were 92.0% and 97.5%, respectively.

Using the MIHP study sample as the reference population yielded an age-adjusted rate of high blood mercury (>40 nmol/L) in the E&H Study sample of 8.0% (95% CI: 5.9, 10.8), which remained significantly different from the rate in the MIHP screening sample of 3.9% (3.0, 5.1). For the hair mercury data, the comparable adjusted rate of high hair mercury (>10 nmol/g) in the E&H Study sample was 4.9% (95% CI: 3.6, 6.7), not significantly different from the rate in the MIHP screening sample of 2.5% (1.6, 3.8).

### Comparison to past levels

In Table 2, the proportions of people with hair mercury concentrations in the E&H Study above 12.5 nmol/g are compared to the Dumont *et al*. [1] data, organised by age and sex. The proportion of people with hair mercury exceeding 12.5 nmol/g was significantly lower in the more recent data for both males (p = 0.001–0.006) and females (p = 0.001–0.012) in all age categories.

### Relationship between fish consumption and mercury exposure

In the E&H Study, 84.9% (95% CI: 84.1, 87.7) of all participants (male and non-pregnant females) who completed the traditional food frequency questionnaire reported consuming locally caught fish at least once in the past year. Furthermore, among women aged 15–44 y (childbearing age), 76.5% reported consuming fish at least once in the past year, while 58.8% reported consuming high mercury predatory fish (i.e. pike, burbot, lake trout or walleye). However, only 10.3% of all participants and 3.5% of women of childbearing age ate fish the recommended twice or more times per week. Among women aged 15–44 y, the median consumption frequency was 0.4 times per month for all fish and 0.1 times for high mercury content predatory fish (see Tables S1 and S2).

Approximately 15.8% (95% CI: 11.6%, 19.9%) of women of childbearing age eating predatory fish had elevated blood mercury levels in comparison to 3.6% (95% CI: 1.2%, 6.1%) of women who ate no predatory fish. Approximately 12.5% (95% CI: 8.8%, 16.3%) of women in this age group eating predatory fish had elevated hair mercury levels, in comparison to 5.0% (95% CI:2.1%, 7.8%) of women eating no predatory fish.

The results of the multiple linear regression analysis examining the relationship between blood and hair mercury concentrations and fish consumption among women of childbearing age are summarised in Table 3. Non-consumers constituted the reference group for fish consumption frequency. Eating predatory fish or non-predatory fish was associated with increased blood and hair mercury concentrations.

**Table 3.** Fitted means (95% CI) for blood and hair mercury concentrations by categories of increasing fish consumption among non-pregnant Cree women aged 15–44 y in *Eeyou Istchee* (northern Quebec, Canada).

Monthly frequency of consumption	N	Blood Mercury (R^2^_adj_ = 0.34)	N	Hair Mercury (R^2^_adj_ = 0.32)
		*Fitted geometric mean mercury concentration, nmol/L (95% CI)*		*Fitted geometric mean mercury concentration, nmol/g (95% CI)*
High mercury predatory fish				
0 meals per month	212	3.37 (2.86, 3.96)	212	0.53 (0.44, 0.63)
>0 and ≤1 meals	203	6.66 (5.45, 8.12)	202	1.17 (0.95, 1.48)
>1 meals	95	11.57 (8.73, 15.34)	93	2.38 (2.01, 3.66)
Lower mercury fish				
0 meals per month	186	3.37 (2.86, 3.96)	187	0.53 (0.44, 0.63)
>0 and ≤1 meals	218	6.48 (5.39, 8.09)	217	0.91 (0.74, 1.17)
>1 meals	107	9.20 (7.08, 12.13)	103	1.10 (0.80, 1.48)

## Discussion

Despite certain exceedances of the recommended blood and hair values, our results suggest that most women of childbearing age sampled had mercury levels that were unlikely to represent a serious hazard to the foetus. Health Canada’s current mercury guidance values were calculated by dividing a blood level of prenatal mercury shown to slightly increase the risk of abnormalities in certain neurodevelopmental tests in children by a safety or uncertainty factor of 5. Nevertheless, Health Canada’s recommended limits are higher than those of some other organisations. The US Environmental Protection Agency (EPA) reference dose for methylmercury is 0.1 μg/kg body weight/day, which corresponded to a hair concentration of 1 μg/g (5 nmol/g) [29]. Evidence of methylmercury’s developmental neurotoxicity at low levels led Grandjean & Budtz-Jorgenson (2007) to suggest an adjustment to the EPA guideline that would result in a recommended maximum concentration in hair of 0.58 μg/g (2.9 nmol/g) [30]. Conversely, the Joint FAO/WHO Expert Committee on Food Additives (2003) recommended a higher exposure limit, namely 2.5 μg/g (12.5 nmol/g) in hair, to account for the possible offsetting of methylmercury toxicity by beneficial nutrients found in seafood [31]. In our analysis, using the more sensitive thresholds mentioned would increase the proportion of the study populations considered to have been at risk of negative effects of methylmercury exposure.

Cree women of childbearing age (15–44 y) in the E&H Study had higher blood mercury concentrations (GM: 9.1 nmol/L, 95% CI: 8.2, 10.1) relative to women aged 16–49 y in the Canadian Health Measures Survey (CHMS Cycle 1, 2007–2009). Women in the latter survey [32] had a geometric mean blood mercury concentration of 0.72 μg/L (95% CI: 0.5, 0.94) or 3.6 nmol/L (2.5, 4.7). Unlike the E&H Study, the CHMS included women who were pregnant [32]. Blood mercury concentrations in pregnant women aged 15–44 in the MIHP screening were lower (GM: 4.4 nmol/L, 95% CI: 4.1, 4.7) than the E&H values, and were more similar to the CHMS data. In a study of First Nations people living on-reservation in Ontario, approximately 7% of childbearing-aged women in the Boreal Shield/Subarctic ecozone had hair mercury concentrations greater than Health Canada’s increased risk level of 10 nmol/g [33]. This is comparable to the 8% of James Bay Cree women of childbearing age in the E&H Study (Table 1). In contrast, nearly 10% of Cree women aged 15–44 y had blood mercury concentrations (Table 1) exceeding Health Canada’s 2010 guidelines [22], compared to more than half of Inuit 18–39 y old women in Nunavik, northern Quebec, in 2004 [34].

The proportion of high blood mercury concentrations among non-pregnant women of childbearing age in the E&H Study was significantly higher than among pregnant women in the MIHP screening programme, even after adjusting for the younger ages in the latter group. These results are consistent with findings from the NHANES survey of an American population, which found that mean blood mercury levels were lower in pregnant women than in women who were not pregnant (0.69 μg/L versus 0.82 μg/L, equivalent to 3.45 and 4.10 nmol/L, respectively); this difference remained after adjusting for age [35]. It is possible that the higher rate of high mercury concentrations in non-pregnant James Bay Cree women reflects seasonal patterns in fish consumption, since the E&H Study data collection took place only in the summer, while the MIHP screening was a year-round programme. However, the well-known increase in blood volume (up to 40% [36]), as well as other metabolic and physiological changes during pregnancy may well constitute an alternative if not better explanation.

Our finding of a decrease in the proportion of participants having a total hair mercury concentration above 12.5 nmol/g is consistent with previous studies showing that mercury exposure in the Cree is decreasing. Dumont *et al*. (1998) [1] reported a decrease in the proportion of people with hair mercury exceeding 15 μg/g (75 nmol/g) in the 1988–1993/94 period. Similarly, in pregnant women, median hair mercury decreased during the period 1983–1991 [37]. The rapid dietary transition among the Cree has likely played a role in this decrease, as traditional food sources have been increasingly replaced with market foods [38]

In the E&H Study, increased consumption frequency of fish was associated with increased blood and hair mercury levels even after accounting for the effect of the 4 fish species known to have the highest mercury levels (Table 3). In spite of having higher mercury levels than the general population, the Cree report relatively low consumption of fish. Among childbearing-aged women, consumption of all fish was well below the recommended 2 servings of low mercury fish per week [17]. Serving size may have been an issue.

Traditional food consumption offers many benefits to the Cree. Fish, in particular, is an excellent source of nutrients including vitamins B3 (niacin) and B12 [39]. It is also one of the best food sources of vitamin D, a nutrient that is particularly important in northern communities [39]. Fish also contains more poly-unsaturated fatty acids (PUFAs) compared to other protein sources, and contains n-3 PUFAs that are not found in other foods [39]. In the E&H Study, increasing traditional food consumption was associated with an increase in serum omega-3 fatty acids [40]. Red blood cell (RBC) n-3 long chain PUFAs have been found to be associated with lower blood glucose in the James Bay Cree population [41]. Levels of n-3 PUFAs seen in the Cree may well be too low to provide related cardio-metabolic health benefits [41]. On this basis, an increase in their fish consumption may be warranted in order to reap the benefits of higher PUFA exposure. Tools such as the online Cree geoportal mercury map (http://www.creegeoportal.ca) provide guidance about which fish species to avoid, or catch more frequently, to reduce mercury exposure.

The transition to a sedentary lifestyle and a diet of more processed market foods has been accompanied by an increase in chronic diseases among the James Bay Cree. Obesity is prevalent, with 77% of women and 64% of men in the E&H Study classified as obese [16]. As a result, chronic diseases associated with obesity are also common. Diabetes is one of the most pressing disease issues in this population, and in 2012 affected 21% of those aged 20 and older [42]. Clearly when discussing traditional food as a source of methylmercury, the risks presented by prenatal exposure to mercury must be balanced with the benefits of harvesting and eating traditional foods.

## Conclusions

The results of our study endorse previous recommendations [13,17] to promote traditional Cree foods while advising pregnant women to limit consumption of higher mercury predatory fish. However, eating lower mercury fish species is not always practical or feasible for women to achieve, since not all species are available in each community and season. Additionally, modern fish harvesting methods favour the rod and line technique, which enhances the catching of predatory fish in comparison to traditional harvesting methods using nets. Our study illustrates that recommendations on fish consumption need to be given in a culturally appropriate way, with awareness of the historical context and traditional importance of fish as a staple food in indigenous populations [43]. Our description of the proportion of the population exceeding recommended methylmercury exposure guidelines can be incorporated into future risk management plans. Ongoing monitoring of mercury in both humans and fish is necessary to ensure the effectiveness of public health risk management activities, as fish consumption advice may need to evolve if blood and hair guidance values are revised or if climate changes enhance the availability of mercury in the food chain at northern latitudes [44].
