# Supplementary material for: Blood and hair mercury concentrations among Cree First Nations of Eeyou Istchee (Quebec, Canada): time trends, prenatal exposure and links to local fish consumption
Source: Int J Circumpolar Health. 2018 May 22;77(1):1474706. doi: 10.1080/22423982.2018.1474706 (PMC5965033; doi:10.1080/22423982.2018.1474706)
Supplement: Supplemental Material [file ZICH_A_1474706_SM4563.docx]

**Supplementary Tables**

**Table S1.** Categories of fish considered in the *Nituuchischaayihtitaau Aschii* Environment-and-Health Study questionnaire.

| Category | Food |
| --- | --- |
| *High* *mercury fish* | Walleye |
|  | Pike |
|  | Burbot |
|  | Lake trout |
|  |  |
| *Lower mercury fish* | Speckled trout |
|  | Whitefish |
|  | Sturgeon |
|  | Red or white sucker |
|  | Fish from the ocean |
|  | Fish eggs |
|  | Smoked wild fish |
|  | Other wild fish |
|  | Fish liver |
|  |  |

**Table S2.** Fish consumption frequencies among women of childbearing age (15-44 y; N = 514) and for all participants aged 8 y (N = 1410) and over in the *Nituuchischaayihtitaau Aschii* Multi-Community Environment-and-Health Study.

|  | Consumption frequency (times per month) for women aged 15-44 y | | | Consumption frequency (times per month) for men and women aged > 7 y | | |
| --- | --- | --- | --- | --- | --- | --- |
| *Category* | ***Mean*** | ***Median*** | ***Geomean (consumers only)*** | ***Mean*** | ***Median*** | ***Geomean (consumers only)*** |
| *All fish* | 1.6 | 0.4 | 0.8 (0.7, 0.9) | 3.8 | 1.0 | 1.4 (1.3, 1.5) |
| *High-mercury fish* | 0.7 | 0.1 | 0.5 (0.4, 0.6) | 1.5 | 0.3 | 0.8 (0.7, 0.9) |
| *Lower-mercury fish* | 0.9 | 0.2 | 0.6 (0.5, 0.7) | 2.3 | 0.4 | 0.9 (0.8, 1.0) |
